# Artificial intelligence anxiety, digital well-being, and future career concerns among engineering and information technology students in Jordan

**DOI:** 10.3389/frai.2026.1598741

**Published:** 2026-03-30

**Authors:** Mais AL-Nasa’h, Luae Al-Tarawneh, Ola Alhwayan

**Affiliations:** 1 Department of Counseling and Special Education, The University of Jordan, Amman, Jordan; 2 Department of Communications Engineering, Princess Sumaya University for Technology, Amman, Jordan

**Keywords:** artificial intelligence anxiety, future career concern, digital well-being, engineering students, information technology students, Jordan

## Abstract

**Introduction:**

The rapid advancement of artificial intelligence (AI) is fundamentally transforming educational and employment landscapes, generating increasing psychological concerns among students in technology-intensive fields. This study examines AI-related anxiety, digital well-being, and career uncertainty among engineering and information technology (IT) students, with a focus on their prevalence, interrelationships, and demographic variations.

**Methods:**

A cross-sectional quantitative design was employed using a structured survey administered to 820 undergraduate students from four Jordanian universities. Standardized measures were used to assess AI anxiety, digital well-being, and career-related concerns. Statistical analyses included descriptive statistics, correlation analysis, and group comparisons based on gender and academic discipline.

**Results:**

The findings indicated elevated levels of AI anxiety (*M* = 5.26, SD = 0.32), low levels of digital well-being (*M* = 1.75, SD = 0.20), and moderate levels of career concerns (*M* = 4.07, SD = 0.34). AI anxiety was strongly negatively correlated with digital well-being (*r* = −0.849, *p* < 0.01) and positively correlated with career concerns (*r* = 0.680, *p* < 0.01). Female students reported significantly higher AI anxiety and career concerns than male students (*p* < 0.001). Additionally, IT students exhibited higher levels of AI anxiety and career uncertainty compared to engineering students (*p* < 0.001).

**Discussion:**

These findings highlight the psychological impact of AI integration on students, emphasizing the need for targeted AI literacy programs, digital well-being interventions, and career guidance strategies. Addressing gender disparities and discipline-specific differences is essential to enhance students’ resilience, adaptability, and readiness for an AI-driven labor market.

## 1 Introduction

Artificial intelligence (AI) is no longer a technology of the distant future; it is actively reshaping higher education, labor markets, and societal expectations. For students in engineering and information technology (IT) programs, this rapid transformation brings substantial opportunity alongside heightened psychological uncertainty. One of the subtler yet consequential challenges is AI anxiety: The apprehension that emerging intelligent systems may threaten one’s competence, professional identity, or future career stability (Chen et al., 2024; Klimova, 2025). This anxiety is particularly salient when students recognize the rise of automation and its implications for human employment.

At the same time, digital well-being—the ability to sustain healthy functioning and balanced boundaries under pervasive connectivity and digitally mediated demands—has become a key concern in contemporary educational settings (Chen et al., 2025; Heiser et al., 2025). Even when students are highly digitally competent, “always-on” academic routines can lead to prolonged screen exposure, reduced recovery time, and blurred boundaries between studying and resting. Evidence also suggests that when digital engagement becomes dysregulated or environmentally unsupported (e.g., poor design, limited inclusivity, or inadequate privacy protections), students’ well-being and learning experiences can deteriorate (Chen et al., 2025; Heiser et al., 2025).

The third domain—future career concerns—captures students’ anticipatory stress about employability, skill relevance, and the formation of their career identities amid rapid structural changes. Evidence from the global labor market indicates accelerated skill transitions and growing expectations for adaptability and continuous learning, which can heighten uncertainty for students preparing for AI-driven workforces (World Economic Forum, 2025). Although AI anxiety, digital well-being, and future career concerns are each receiving increasing attention, empirical research that integrates these constructs within a single explanatory framework remains limited. This gap appears even more pronounced in non-Western contexts and among STEM students, where AI exposure is intensive and employability transitions are particularly salient (World Economic Forum, 2025; Wu and Li, 2025; Cao et al., 2026). Accordingly, an integrative model is needed to clarify how AI-related anxiety relates to coping-related digital well-being and long-term future career concerns (Wu and Li, 2025).

In Jordan, national policy places digital transformation and AI adoption at the center of economic modernization. The Ministry of Digital Economy and Entrepreneurship (MoDEE) has released an AI Strategy and Implementation Plan (2023–2027), a Digital Inclusion Policy (2025), and a Digital Transformation Strategy (2026–2028), signaling an accelerated expansion of AI-enabled services and heightened expectations for skill development [Ministry of Digital Economy and Entrepreneurship (MoDEE), 2021; Ministry of Digital Economy and Entrepreneurship (MoDEE), 2023; Ministry of Digital Economy and Entrepreneurship (MoDEE), 2025; Ministry of Digital Economy and Entrepreneurship (MoDEE), 2026]. These pressures are particularly salient in Jordan, where official statistics indicate persistently high unemployment rates (21.3% in the first quarter of 2025), intensifying students’ sensitivity to perceived career instability in AI-driven labor markets (Department of Statistics (Jordan), 2025). Taken together, these socioeconomic and educational conditions help explain why STEM students may perceive AI-driven changes as a career-relevant threat unless their coping resources and career adaptability are strengthened (World Economic Forum, 2025). Accordingly, this study addresses the following questions:

1. What are the levels of AI anxiety, digital well-being, and future career concerns among engineering and IT students in Jordan?
2. How are these constructs statistically and conceptually interrelated (*p* ≤ 0.05)?
3. To what extent do gender and academic discipline influence these perceptions and outcomes?

By testing an integrative AI–Career Stress Model, the study aims to inform interventions that combine AI literacy, digital well-being support, and career guidance strategies for STEM students navigating AI-driven transitions.

## 2 Literature review

Recent synthesis research (2025–2026) shows that students’ responses to AI in higher education are not purely cognitive; they include appraisal-based anxiety reactions that shape engagement and well-being (Klimova, 2025; Wu and Li, 2025). In parallel, systematic evidence on digital well-being emphasizes that self-regulation, emotional balance, and boundary management distinguish adaptive digital engagement from maladaptive dependency, with implications for learning and future orientation (Chen et al., 2025; Heiser et al., 2025; Steele et al., 2020; Odgers and Jensen, 2020).

### 2.1 AI anxiety in higher education

As AI tools become commonplace in learning, assessment, and career preparation, AI anxiety has emerged as a recognizable form of technology-linked apprehension in higher education (Wang and Wang, 2019; Wu and Li, 2025; He et al., 2025; Abdallat et al., 2025). AI anxiety is often triggered when students interpret AI as a competence threat, an identity threat, or a future threat—particularly in technology-intensive disciplines where automation is perceived as directly relevant to academic performance and employability (World Economic Forum, 2025; Wu and Li, 2025; Abdallat et al., 2025). A recent systematic review synthesizing the education literature reported consistent links between AI anxiety and outcomes such as behavioral intention, self-efficacy, and learning-related responses, while also calling for the development of integrative models and mitigation frameworks to address these concerns (Wu and Li, 2025).

Importantly, AI anxiety does not necessarily translate into avoidance. In many higher education settings, students may continue using AI tools despite apprehension, particularly when AI use is structurally encouraged or perceived as unavoidable. Recent evidence indicates that AI anxiety can co-exist with adoption intentions and may influence these intentions through intertwined perceptions of usefulness, risk, and trust (Cao et al., 2026). In parallel, empirical research also shows that AI-mediated learning environments can be associated with anxiety and related emotional outcomes in learners, underscoring that these reactions are not merely theoretical but observable in practice (He et al., 2025). Moreover, AI anxiety has been linked to meaningful learning-related responses, supporting the view that anxiety may influence how students engage rather than merely whether they adopt AI (Chen et al., 2024).

From a measurement perspective, the Artificial Intelligence Anxiety Scale (AIAS) operationalizes AI anxiety as multidimensional—capturing performance concerns, job displacement fears, societal impact anxieties, and discomfort in human–AI interaction—thereby supporting more precise testing of how specific facets relate to learning and future-oriented outcomes (Wang and Wang, 2019).

### 2.2 Digital well-being as a coping-related resource

Digital well-being has become central to understanding how students function in “always-on” learning environments. It reflects the extent to which individuals can regulate digital demands, maintain healthy boundaries, and preserve psychological recovery, rather than merely demonstrating digital skill (Chen et al., 2025). Recent evidence further argues that digital well-being should be analytically distinguished from digital competence: Students may be highly capable users while still experiencing dysregulated engagement, weakened boundaries, and reduced recovery (Chen et al., 2025; Heiser et al., 2025). More broadly, research on mental health in the digital age emphasizes that outcomes are shaped by patterns of use and contextual conditions—not by exposure alone—supporting the need to examine how students manage and sustain their digital practices over time (Odgers and Jensen, 2020). In educational settings, digital well-being is also influenced by learning-environment design choices, including inclusion- and privacy-conscious practices in online and video conferencing contexts (Heiser et al., 2025).

This view aligns with broader research on mental health in the digital age, which argues that digital outcomes are shaped by patterns of use and contextual conditions rather than exposure alone (Odgers and Jensen, 2020), and with digital stress models suggesting that stress processes can mediate the link between persistent digital demands and psychosocial functioning (Steele et al., 2020). Complementing this perspective, a systematic review on equitable video conferencing learning indicated that inclusion, privacy, and instructional design choices materially influence students’ digital well-being experiences, reinforcing that digital well-being is shaped by both individual resources and the learning environment (Heiser et al., 2025). Taken together, this literature supports conceptualizing digital well-being as a coping-related resource that may buffer technology-related stressors and limit the spillover of AI-related threat appraisals into broader distress and insecurity (Chen et al., 2025; Heiser et al., 2025; Steele et al., 2020).

### 2.3 Future career concern under conditions of AI-driven labor market change

Future career concerns refer to students’ anticipatory stress about employability, perceived skill obsolescence, and the development of a coherent professional identity under conditions of rapid economic and technological change. These concerns emerge when individuals question whether their current competencies will remain relevant and whether they can successfully navigate increasingly fluid career trajectories (Tsai et al., 2017; Savickas, 2005).

Global labor market analyses consistently highlight accelerated skill transitions, the reconfiguration of job roles, and rising expectations for adaptability and lifelong learning, all of which intensify uncertainty for students preparing to enter AI-influenced workplaces. Importantly, these transitions are not limited to job displacement but increasingly emphasize reskilling and upskilling, with employers prioritizing learning agility and the ability to collaborate with intelligent systems (World Economic Forum, 2025).

Within STEM disciplines, future career concerns may be especially pronounced. Early and intensive exposure to AI-enabled workflows can increase students’ awareness of automation-related change, leading them to perceive greater instability in entry-level roles and less predictable pathways for early career progression (World Economic Forum, 2025; Savickas, 2005). From a career construction perspective, such uncertainty can disrupt future orientation and weaken career adaptability resources, thereby intensifying concerns about long-term professional viability (Savickas, 2005).

### 2.4 Integrative gap and theoretical bridge

Although research on AI anxiety, digital well-being, and future career concerns is expanding, these bodies of research largely develop in parallel rather than being integrated within a single explanatory framework. Recent synthesis research in education highlights the need to move beyond isolated associations by clarifying how AI-related anxiety connects to downstream educational and vocational outcomes through plausible mechanisms and contextual conditions (Wu and Li, 2025). In parallel, contemporary research on digital well-being emphasizes that students’ functioning in technology-saturated environments is shaped not only by digital competence but also by sustainable self-regulation, boundary management, and psychological recovery—features that distinguish adaptive engagement from dysregulated dependency patterns (Chen et al., 2025; Heiser et al., 2025). Taken together, this points to a clear gap: Empirical research that simultaneously examines how AI anxiety relates to coping-related digital well-being and, in turn, to future career concerns remains limited, particularly among STEM students in non-Western higher education contexts, where AI exposure is both curricular and directly tied to employability expectations (World Economic Forum, 2025; Wu and Li, 2025; Abdallat et al., 2025).

To address this gap, the present study proposes an integrative AI–Career Stress Model grounded in Lazarus’s stress appraisal and coping framework and Savickas’s career construction perspective (Lazarus, 1966; Savickas, 2005). From a stress appraisal standpoint, AI anxiety can be conceptualized as an appraisal-based response in which the primary appraisal frames AI-driven change as a potential threat (e.g., perceived competence erosion, displacement, or uncertainty about professional role identity), while the secondary appraisal reflects perceived coping resources and the extent to which students feel capable of managing these demands (Lazarus, 1966). Within this framework, digital well-being is treated as a proximal, coping-related resource that captures the capacity to regulate digital demands, maintain boundaries, and support psychological recovery under conditions of continuous connectivity (Chen et al., 2025; Heiser et al., 2025). Complementary research on digital stress further supports this mechanism by describing how stress processes can mediate the relationship between digitally driven demands and psychosocial functioning (Steele et al., 2020).

From a career construction perspective, sustained threat appraisals and diminished coping resources may reduce career adaptability and future-oriented agency, thereby intensifying future career concerns (Savickas, 2005). In practical terms, students who experience persistent AI anxiety while struggling to sustain balanced digital functioning may be more likely to anticipate skill obsolescence, doubt employability, and perceive fewer viable pathways for constructing a stable career trajectory in an AI-shaped labor market (World Economic Forum, 2025; Savickas, 2005). Therefore, this integrative framing positions digital well-being as a key explanatory link connecting AI anxiety to future career concerns, while recognizing that the strength of these pathways depends on broader structural conditions.

Context is particularly relevant in Jordan, where national policy explicitly prioritizes digital transformation and AI adoption as part of economic modernization. Official policy documents outline an ambitious roadmap for AI diffusion, capacity building, and digital inclusion, signaling heightened skills expectations for emerging graduates (Ministry of Digital Economy and Entrepreneurship (MoDEE), 2021; Ministry of Digital Economy and Entrepreneurship (MoDEE), 2023; Ministry of Digital Economy and Entrepreneurship (MoDEE), 2025; Ministry of Digital Economy and Entrepreneurship (MoDEE), 2026). Concurrently, labor market pressures remain salient, as reflected in official unemployment indicators (Department of Statistics (Jordan), 2025). These intersecting conditions make Jordan a theoretically informative setting for examining how STEM students appraise AI-driven change, mobilize coping-related digital well-being resources, and translate these experiences into future career outlooks [World Economic Forum, 2025; Ministry of Digital Economy and Entrepreneurship (MoDEE), 2021; Ministry of Digital Economy and Entrepreneurship (MoDEE), 2023; Ministry of Digital Economy and Entrepreneurship (MoDEE), 2025; Ministry of Digital Economy and Entrepreneurship (MoDEE), 2026; Department of Statistics (Jordan), 2025].

**Figure 1:**
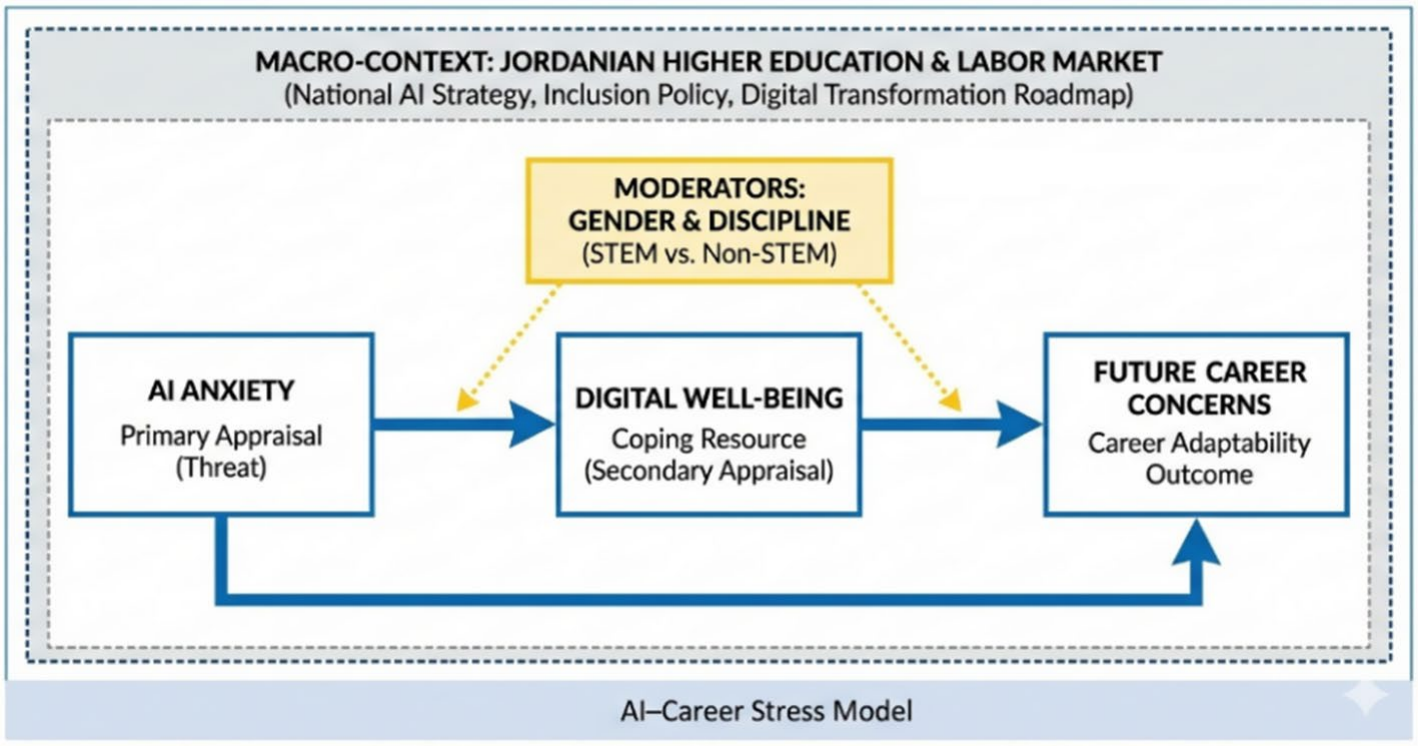
AI-career stress model.

Accordingly, Figure 1 presents the conceptual schema: AI anxiety is expected to predict future career concerns both directly and indirectly through digital well-being, with gender and academic discipline included as moderators and the model interpreted within Jordan’s policy and labor market context [World Economic Forum, 2025; Wu and Li, 2025; Ministry of Digital Economy and Entrepreneurship (MoDEE), 2021; Ministry of Digital Economy and Entrepreneurship (MoDEE), 2023; Ministry of Digital Economy and Entrepreneurship (MoDEE), 2025; Ministry of Digital Economy and Entrepreneurship (MoDEE), 2026; Department of Statistics (Jordan), 2025; Lazarus, 1966; Savickas, 2005].

## 3 Methodology

### 3.1 Study design

This study employed a quantitative, cross-sectional survey design to examine levels of AI anxiety, digital well-being, and future career concerns among engineering and IT students in Jordan. Ethical approval was obtained from the Research Ethics (IRB) Committee, Faculty of Educational Sciences. The study protocol was reviewed and approved (Protocol No. 3842/2025/21, 10/02/2025). All participants provided electronic informed consent before completing the survey, and responses were collected anonymously.

Data were collected in February 2025 using an online structured questionnaire administered through a secure, university-hosted platform. Participation was voluntary and anonymous, and the survey settings were configured to allow only a single submission per participant to ensure data integrity.

An *a priori* power analysis (*α* = 0.05; 1 − *β* = 0.80) was conducted to ensure adequate statistical power for the planned multivariate comparisons (multivariate analysis of variance (MANOVA)). The analysis estimated a minimum required sample of 650 participants to detect a medium effect. The final analytic sample (*N* = 820) exceeded this threshold, indicating sufficient power for the intended analyses.

### 3.2 Participants

The study included 820 undergraduate students recruited from four Jordanian universities. These universities were purposefully selected to ensure representation across public and private institutions, geographic regions, and variation in engineering and IT curricula. Participants were recruited using convenience sampling through institutional mailing lists and student groups. The online survey required approximately 10 min to complete.

**Figure 2:**
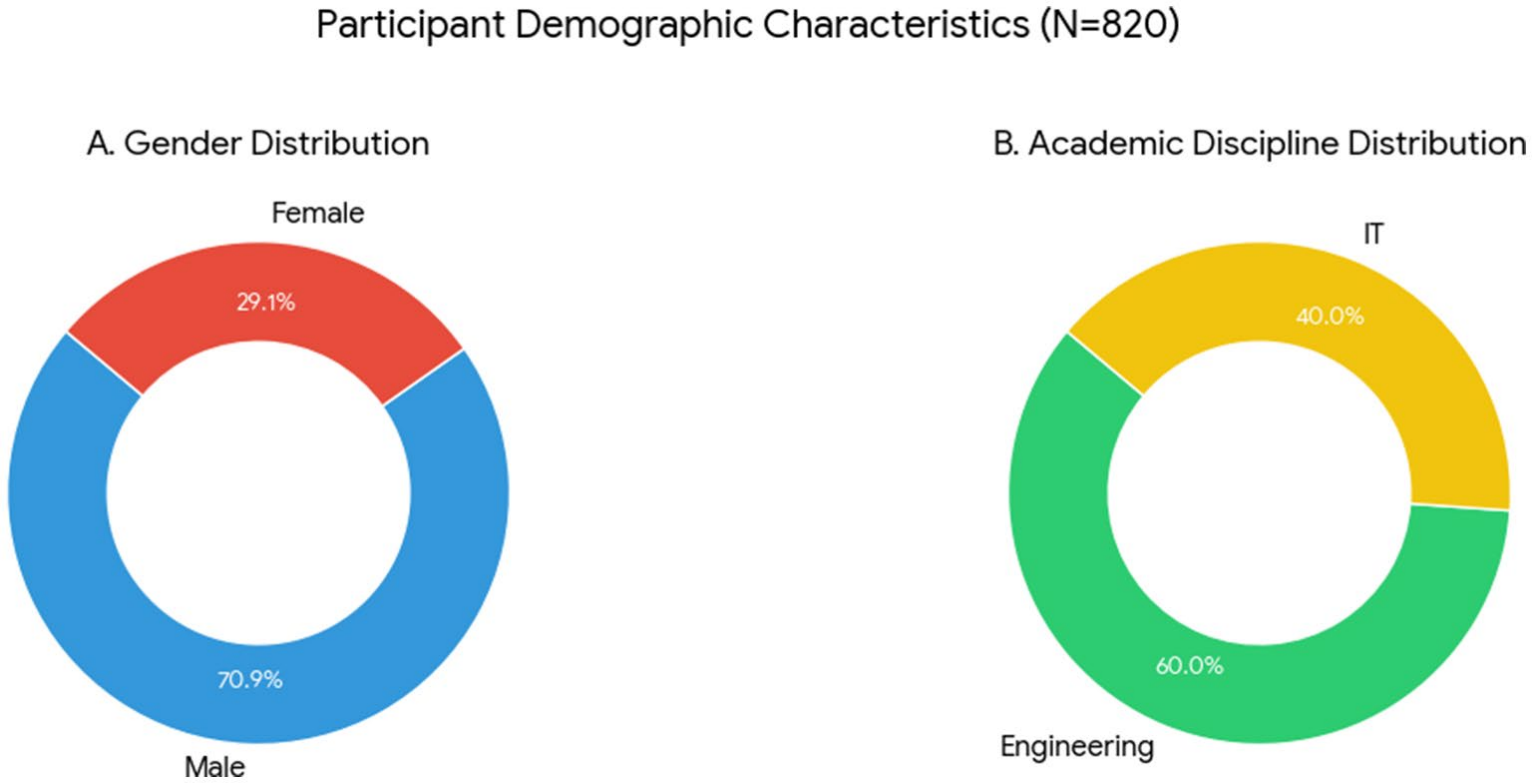
Participant demographic characteristics (*N* = 820).

A total of 873 responses were initially received. After screening the data for completeness, internal consistency, and attention-check performance, 820 responses were retained for analysis. In the final sample, 581 participants (70.9%) were male students and 239 (29.1%) were female students. By academic discipline, 492 students (60.0%) were enrolled in engineering programs and 328 (40.0%) were enrolled in IT programs (see Figure 2).

Before participation, students received a clear explanation of the study’s purpose and procedures and provided electronic informed consent. The responses were anonymous, voluntary, and uncompensated, and all procedures adhered to applicable ethical standards. Participant characteristics are summarized in Table 1.

**Table 1:** Characteristics of samples (*N* = 820).

Academic field	Male	Female	Total
IT	296	32	328
Engineering	285	207	492
Total	581	239	820

### 3.3 Instrumentation

#### 3.3.1 AI anxiety scale (AIAS)

The Artificial Intelligence Anxiety Scale (AIAS), developed and validated by Wang and Wang, was used to assess AI-related anxiety among university students. The instrument consists of 21 items organized into four interrelated dimensions: Performance anxiety (concerns about AI outperforming human capability), societal impact anxiety (worries about broader social consequences), job displacement anxiety (perceived employment threats), and interaction anxiety (discomfort when engaging with AI systems). The items are rated on a 7-point Likert scale ranging from 1 (Strongly Disagree) to 7 (Strongly Agree) (Wang and Wang, 2019).

To ensure contextual appropriateness for Jordanian students, the AIAS was translated into Arabic using forward translation and independent back-translation by bilingual experts, followed by reconciliation and cognitive debriefing with five students to confirm clarity and semantic equivalence. A panel of 10 experts in educational psychology and AI education reviewed the Arabic version for clarity and cultural relevance (agreement = 90%). A pilot study (*n* = 40) confirmed the feasibility and internal consistency (Cronbach’s *α* > 0.80 across dimensions).

The four-factor structure was retained, in line with the original theoretical model proposed by Wang and Wang (2019). The use of validated AI anxiety measures is consistent with recent higher education research examining anxiety-related responses in AI-mediated learning contexts (He et al., 2025; Murakami and Inagaki, 2025).

#### 3.3.2 Digital well-being scale (DWBS)

Digital well-being was measured using the Digital Well-Being Scale (DWBS) developed and psychometrically validated by Arslankara et al. (2022). The DWBS comprises 12 items across 3 subscales—digital satisfaction, safe and responsible behavior, and digital wellness—and the responses were recorded on a 5-point Likert scale ranging from 1 (Strongly Disagree) to 5 (Strongly Agree) (Arslankara et al., 2022). The DWBS was selected because it captures both hedonic and eudaimonic aspects of digital engagement, making it conceptually relevant for examining stress-related technology experiences and self-regulation in digitally intensive learning environments (Chen et al., 2025; Odgers and Jensen, 2020).

In this study, the DWBS was translated and culturally adapted using forward translation and independent back-translation procedures, followed by reconciliation to ensure linguistic and conceptual equivalence. In total, 10 bilingual experts reviewed item wording for clarity and cultural suitability (≥ 90% agreement), and a pilot test (*n* = 40) confirmed comprehensibility and response consistency. In the present sample, psychometric evaluation supported the intended three-factor structure (CFI = 0.95; RMSEA = 0.04) and demonstrated strong internal consistency (Cronbach’s *α* = 0.86; Spearman–Brown split-half = 0.84).

#### 3.3.3 Career future concern scale (CFCS)

Career future concern was assessed using an adapted short form of the career anxiety scale developed and validated by Tsai et al. (2017). The adapted measure comprises five items targeting anticipatory concerns about employability, skill relevance, and career stability in the context of AI-driven labor market change. Items are rated on a 5-point Likert scale (1 = Strongly Disagree to 5 = Strongly Agree). To ensure content relevance for STEM students in Jordan, the adapted items were reviewed by a panel of 10 specialists in career counseling and industrial engineering (agreement ≥ 90%). A pilot test (*n* = 40) confirmed the clarity and internal consistency (Cronbach’s *α* = 0.89). In the final sample, item–total correlations ranged from 0.49 to 0.90. A confirmatory factor analysis (CFA) supported a single-factor structure, showing satisfactory discriminant validity in relation to AI anxiety and digital well-being.

### 3.4 Data analysis

Data were analyzed using IBM SPSS Statistics (Version 26) (IBM Corp., 2019). Before inferential testing, the dataset was screened for accuracy and quality, including checks for missing values, univariate outliers, and distributional assumptions using Q–Q plots and skewness–kurtosis indices. Descriptive statistics were used to summarize levels of AI anxiety, digital well-being, and future career concern. Pearson correlation coefficients were computed to examine bivariate associations among the study variables. Group differences by gender and academic discipline were evaluated using MANOVA, which is appropriate for examining multiple correlated dependent variables simultaneously. The assumptions for MANOVA were evaluated before interpretation, including homogeneity of error variances (Levene’s test) and equality of covariance matrices (Box’s M test), as implemented in SPSS (Stöhr et al., 2024; Cronbach, 1951; Field, 2018).

To complement statistical significance testing, effect sizes were reported for all relevant comparisons, including Cohen’s d and partial η^2^, following standard conventions for interpreting practical significance in the behavioral sciences. In addition, variance inflation factor (VIF) values were examined to evaluate multicollinearity, with all values remaining below the commonly accepted conservative threshold, indicating no problematic redundancy among predictors. Reliability was assessed using Cronbach’s *α*, and values exceeded 0.80 for all scales, supporting internal consistency (Cronbach, 1951).

Finally, to assess measurement quality, CFA was conducted using maximum-likelihood estimation, and model fit was evaluated using widely established criteria and reporting practices for indices such as χ^2^/df, CFI, and RMSEA (Cohen, 1988). All hypothesis tests were conducted at α = 0.05, and 95% confidence intervals were reported where appropriate. Finally, confirmatory factor analysis (CFA) was performed using maximum-likelihood estimation to assess measurement quality. Model fit was evaluated based on established indices (χ^2^/df, CFI, and RMSEA), in line with recommended structural equation modeling guidelines (Kline, 2016). Statistical significance was set at α = 0.05, with 95% confidence intervals reported where applicable.

## 4 Results

As artificial intelligence (AI) continues to transform education and employment, understanding its psychological and professional effects on students in technology-focused disciplines is crucial. This section presents descriptive, correlational, and inferential analyses that together clarify how AI anxiety, digital well-being, and future career concerns interact within the proposed AI–Career Stress Model.

### 4.1 Descriptive statistics

Data from 820 students in engineering and IT programs were analyzed. Mean scores showed that AI anxiety was high (*M* = 5.26, SD = 0.32, range 1–7), indicating broad apprehension about automation, algorithmic decision-making, and potential job displacement. Digital well-being was low (*M* = 1.75, SD = 0.20, range 1–5), suggesting difficulty in maintaining a balance between online engagement and mental health. Future career concerns was moderate to high (*M* = 4.07, SD = 0.34, range 1–5), reflecting uncertainty about employability and skill relevance in an AI-driven labor market.

All variables were approximately normally distributed (skewness and kurtosis < ±1). Confidence intervals confirmed the stability of the means (AI anxiety [5.23, 5.29]; digital well-being [1.73, 1.77]; career concern [4.05, 4.10]). Together, these data indicate that heightened anxiety about AI corresponds with lower digital well-being and greater apprehension about future careers. This pattern aligns with the AI–Career Stress Model, in which technological anxiety indirectly undermines career confidence by diminishing digital balance (Table 2).

**Table 2:** Descriptive statistics for study variables (*N* = 820).

Variable	*N*	Mean	SD
AI Anxiety	820	5.26	0.32
Digital Well-Being	820	1.75	0.20
Future- Career Concern	820	4.07	0.34

### 4.2 Correlational relationships

**Figure 3:**
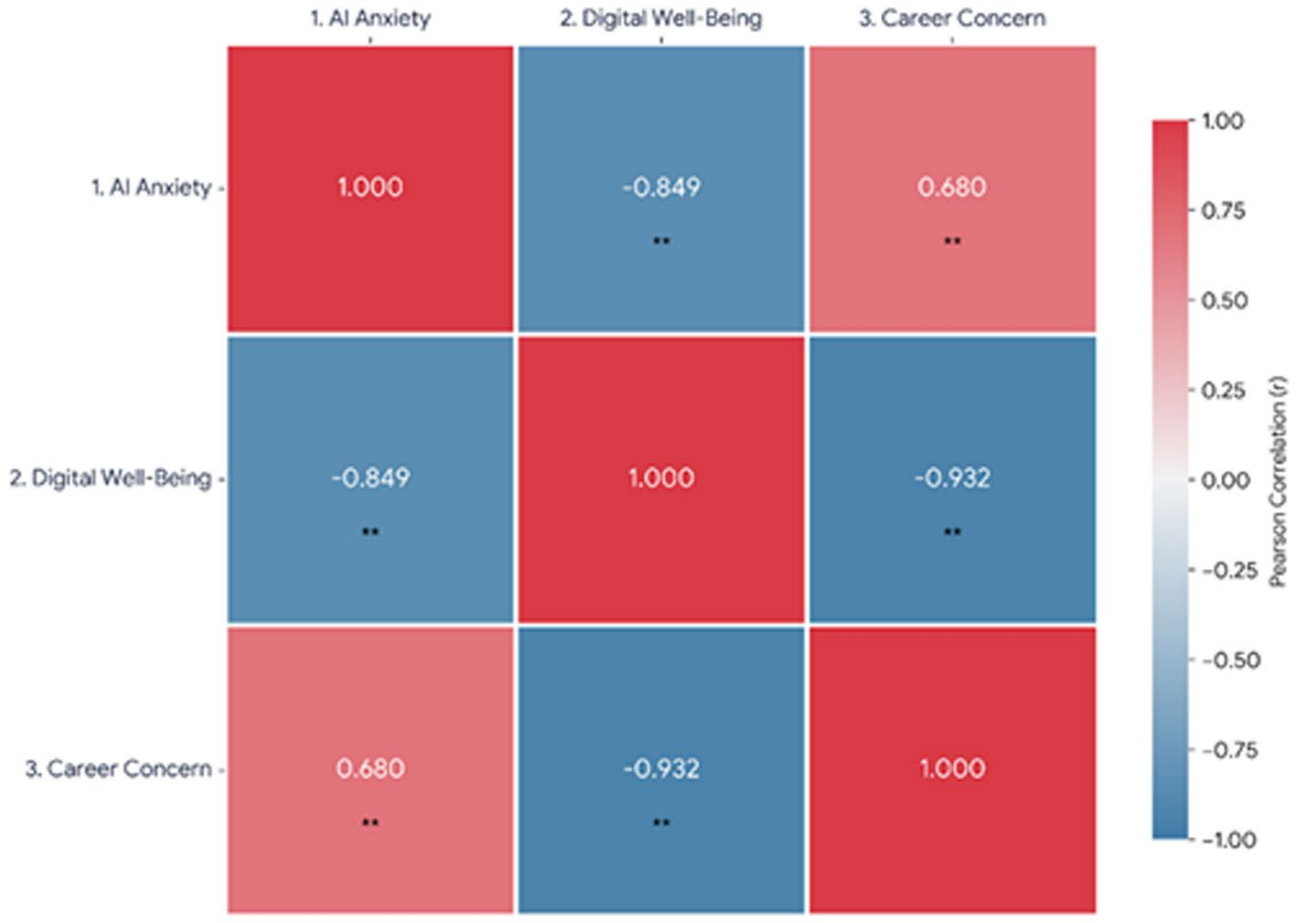
Inter-variable correlation matrix. *N* = 820; *p* < 0.01 (two-tailed).

Pearson correlations (Figure 3) demonstrated strong, theoretically consistent relationships among variables. AI anxiety and digital well-being were strongly and negatively correlated (*r* = −0.849, *p* < 0.001, 95% CI [−0.87, −0.82]), showing that greater fear of AI is associated with poorer digital regulation. AI anxiety and future career concerns were positively correlated (*r* = 0.680, *p* < 0.001, 95% CI [0.64, 0.72]), suggesting that anxiety about automation translates into insecurity regarding professional identity and employment. Digital well-being and future career concerns were negatively correlated (*r* = −0.932, *p* < 0.001, 95% CI [−0.94, −0.92]), indicating that students with better digital balance reported fewer career-related concerns. Assumption testing confirmed linearity and homoscedasticity, and Variance Inflation Factor values below 2 verified the absence of multicollinearity. The observed pattern of relationships supports the AI–Career Stress Model: Higher AI anxiety undermines digital well-being, which, in turn, exacerbates career-related concerns.

### 4.3 Gender differences

These results are consistent with broader evidence that women can face structural barriers and underrepresentation in technology-intensive pathways during periods of rapid technological change, which may heighten anxiety and career uncertainty. Such patterns have been discussed in global workforce analyses highlighting widening skills transitions and uneven access to emerging opportunities, as well as in reports focusing on how AI-driven transformations can differentially affect women’s working lives (World Economic Forum, 2025; Collett et al., 2022). These results align with evidence showing systematic gender differences in students’ perceptions and use of AI tools in higher education, where female students often report greater concerns and less positive attitudes toward AI applications compared to male students. Such patterns have been observed in large, multi-university samples and are frequently discussed in relation to structural barriers, underrepresentation, and gendered experiences in technology-intensive fields (Collett et al., 2022; Stöhr et al., 2024) (see Table 3).

**Table 3:** Tests of between- subjects effects (MANOVA).

Source	Dependent variable	*F*	Sig.	Partial η^2^
Gender	AI Anxiety	42.08	< 0.001	0.049
	Digital Well-Being	3.63	0.057	0.004
	Future career concerns	41.32	< 0.001	0.048
Discipline	AI Anxiety	27.35	< 0.001	0.032
	Digital Well-Being	29.26	< 0.001	0.035
	Future Career Concern	29.99	< 0.001	0.035

### 4.4 Disciplinary differences

A second MANOVA examining academic discipline showed a significant overall effect, Wilks’ *Λ* = 0.96, *F*(3, 816) = 10.89, *p* < 0.001, partial η^2^ = 0.04. IT students reported higher AI anxiety [*F*(1, 817) = 27.35, *p* < 0.001, η^2^ = 0.032] and greater future career concerns [*F*(1, 817) = 29.99, *p* < 0.001, η^2^ = 0.035] than engineering students, whereas engineering students scored higher on digital well-being [*F*(1, 817) = 29.26, *p* < 0.001, η^2^ = 0.035] (see Table 3).

This pattern may reflect differences in perceived proximity to AI-driven task automation and skill disruption. For example, global labor market evidence indicates that AI is accelerating changes in job tasks and increasing expectations for reskilling and adaptability, which may be appraised as a greater employability threat in AI-intensive domains (World Economic Forum, 2025). The gender × discipline interaction was not significant (*p* > 0.05), indicating that gender patterns were consistent across both fields.

### 4.5 Integrated interpretation

The quantitative results outline a clear psychological profile of Jordanian STEM students in the context of ongoing digital transformation. High AI anxiety combined with low digital well-being and moderate future career concerns reflects a meaningful tension between enthusiasm for innovation and apprehension about automation. The strong associations among these constructs suggest that digital balance may operate as a protective resource, helping to buffer the emotional cost of rapid technological change.

Reliability estimates indicated strong internal consistency across measures (all Cronbach’s *α* > 0.80). Confirmatory factor analysis further supported the adequacy of the measurement model (CFI = 0.95; RMSEA = 0.04), providing evidence of satisfactory construct validity within the present sample. Collectively, these results lend empirical support to the AI–Career Stress Model: Higher AI anxiety is associated with reduced digital well-being, which, in turn, is linked to elevated concerns about career prospects. The findings underscore the value of integrated educational strategies that strengthen AI literacy, promote digital well-being skills, and support psychological resilience among students preparing for AI-driven transitions.

## 5 Discussion

The findings of this study indicate that Jordanian university students—particularly those enrolled in technology-focused disciplines—experience high levels of AI-related anxiety, low digital well-being, and moderate to high future career concerns. These results provide a clear psychological profile of students navigating rapid digital transformation: A generation both inspired by AI’s potential and unsettled by its implications for their personal and professional futures.

### 5.1 AI anxiety in context

The elevated AI anxiety observed among engineering and IT students is consistent with evidence showing that AI-related apprehension is increasingly documented in higher education settings and is associated with learning- and behavior-relevant outcomes (Wang and Wang, 2019; Wu and Li, 2025). Broader research on AI and student well-being in higher education further supports the view that students’ responses to AI adoption can include meaningful psychological reactions that extend beyond purely technical considerations (Klimova, 2025).

In parallel, global labor market analyses highlight accelerated skill transitions, task reconfiguration, and increasing reskilling and upskilling pressures, which can amplify students’ uncertainty about employability and professional identity in AI-shaped economies (World Economic Forum, 2025). Within Jordan, national digital transformation and AI policy initiatives signal an intensified push toward AI readiness and the diffusion of AI-enabled services, while official indicators continue to reflect labor market pressures [Ministry of Digital Economy and Entrepreneurship (MoDEE), 2021; Ministry of Digital Economy and Entrepreneurship (MoDEE), 2023; Ministry of Digital Economy and Entrepreneurship (MoDEE), 2025; Ministry of Digital Economy and Entrepreneurship (MoDEE), 2026; Department of Statistics (Jordan), 2025]. Taken together, these conditions may help explain why AI-related anxiety in this context can be experienced as a future-oriented stressor linked to perceived employability risk and readiness demands (World Economic Forum, 2025; Wu and Li, 2025; Abdallat et al., 2025).

### 5.2 Gender disparities and socio-cultural barriers

Consistent with prior evidence that gendered differences can shape how students engage with emerging AI tools, female students in our sample reported significantly higher AI anxiety and greater future career concerns than male students. This pattern aligns with recent higher education research documenting meaningful variation across genders in perceptions and the use of AI chatbots and related tools (Stöhr et al., 2024). In the Middle East and North Africa, gender disparities in STEM participation and progression have also been repeatedly linked to structural and sociocultural barriers (e.g., access to mentoring, field norms, and opportunity pipelines), which may intensify technology-related anxiety and future-career uncertainty (Cohen, 1988).

Moreover, broader policy reports on AI and women’s work highlight that technology transitions can reproduce or amplify existing inequities in access, advancement, and job security—making “AI-driven displacement” concerns especially salient for women in male-dominated sectors (Collett et al., 2022). Collectively, these considerations suggest that AI anxiety is not purely an individual reaction; it can reflect unequal opportunity structures that shape confidence, participation, and belonging in AI-adjacent fields.

### 5.3 Digital well-being and psychological resilience

The finding that students reported low digital well-being aligns with prior literature highlighting the psychological toll of excessive digital exposure and cognitive overload in technology-intensive environments (Steele et al., 2020; Odgers and Jensen, 2020; Lazarus, 1966). For students immersed in AI-assisted learning and constant connectivity, mental strain and screen fatigue are becoming increasingly prevalent. Empirical evidence indicates that digital exhaustion is associated with heightened anxiety and burnout, which can impair focus, motivation, and self-efficacy (Arslankara et al., 2022). Complementing this view, technostress research demonstrates that persistent technology-enabled demands can produce cumulative cognitive and emotional strain, negatively affecting well-being and performance when coping resources are insufficient (Califf et al., 2020).

Notably, intervention-oriented studies suggest that structured digital mindfulness programs—emphasizing time management, reflective screen use, and balanced digital engagement—can effectively enhance students’ psychological resilience and cognitive recovery (Chen et al., 2023).

Within the Jordanian context, where AI-enabled platforms are progressively embedded in higher education (Wu and Li, 2025), universities must move beyond a sole focus on technical skill development to address the human dimensions of digital engagement. Integrating digital wellness workshops, counseling support, and workload management strategies may foster emotional regulation, sustain digital balance, and strengthen academic persistence among students navigating AI-driven learning environments.

### 5.4 Career anxiety and labor market adaptation

The low digital well-being observed in this study is consistent with prior evidence showing that students’ mental health outcomes in digital environments depend more on patterns of use, self-regulation, and contextual demands than on digital exposure alone (Steele et al., 2020; Odgers and Jensen, 2020). In technology-intensive learning contexts—where students are continuously connected and increasingly rely on AI-enabled tools—persistent digital demands can accumulate into digital stress and hinder psychological recovery, which may undermine attention, motivation, and academic functioning (Steele et al., 2020; Odgers and Jensen, 2020). In addition, empirical research indicates that higher levels of digital health literacy are associated with more adaptive information use and better well-being among university students, suggesting that strengthening students’ digital coping competencies can support resilience under intensive digital conditions (Chen et al., 2023). Taken together, these findings reinforce the interpretation that digital well-being functions as a coping-related resource within the proposed model, potentially shaping how AI-related stress experiences translate into broader academic and future-oriented adjustment (Steele et al., 2020; Odgers and Jensen, 2020; Chen et al., 2023).

Within Jordan, where digital transformation and AI adoption are being rapidly advanced at the national level (Ministry of Digital Economy and Entrepreneurship (MoDEE), 2023; Ministry of Digital Economy and Entrepreneurship (MoDEE), 2025; Ministry of Digital Economy and Entrepreneurship (MoDEE), 2026), universities may benefit from complementing AI literacy with structured support that promotes healthy digital routines (e.g., boundary setting, recovery time, and skills for managing information demands) to protect students’ well-being and sustain academic engagement (Steele et al., 2020; Odgers and Jensen, 2020; Chen et al., 2023).

### 5.5 Integrative perspective

The interrelations identified in this study substantiate the proposed AI–Career Stress Model, illustrating a clear psychological pathway: AI anxiety undermines digital well-being, which, in turn, amplifies career insecurity. This pattern is consistent with Lazarus’s stress appraisal and coping framework—in which perceived threat appraisals generate stress responses—and with Savakis’s career construction theory, which highlights how uncertainty can disrupt career adaptability and future-oriented career development (Lazarus, 1966; Savickas, 2005). Taken together, the model provides a theoretically grounded explanation of how cognitive–emotional reactions to AI may translate into reduced digital functioning and heightened concern about career futures in AI-rich educational environments.

### 5.6 Practical implications and future directions

This study offers actionable implications for policymakers and higher education leaders. At the university level, engineering and IT programs should integrate AI literacy and human–AI collaboration competencies while simultaneously strengthening students’ self-regulation and digital well-being skills through structured support (e.g., boundary management, workload planning, and counseling-informed guidance). At the national level, aligning academic preparation with Jordan’s AI strategy and implementation roadmap can facilitate smoother transitions from higher education to AI-shaped labor markets (Ministry of Digital Economy and Entrepreneurship (MoDEE), 2023). In parallel, institutional policies that support equitable and inclusive digital learning environments (e.g., privacy-conscious design and accessibility) may contribute to healthier student digital well-being experiences (Heiser et al., 2025).

Gender inclusivity should remain central to these reforms. Given that women’s experiences in technology-intensive environments can be shaped by structural barriers and differential exposure to AI-related risks, targeted mentorship, visible role models, and supportive networks (e.g., Women in AI initiatives) are recommended to reduce anxiety and strengthen career confidence (Collett et al., 2022; Cohen, 1988).

Finally, future research should adopt longitudinal and mixed methods designs to examine how AI anxiety, digital well-being, and career adaptation develop over time. Qualitative interviews could capture context-specific meanings of “threat,” “coping,” and “career uncertainty” that are not fully observable through cross-sectional survey designs (Lazarus, 1966; Savickas, 2005).

### 5.7 Limitation

Although this study provides valuable insights into the interconnection between AI anxiety, digital well-being, and future career concerns among Jordanian engineering and IT students, several limitations should be acknowledged to guide future research. First, the study employed a cross-sectional design, capturing perceptions at a single point in time. Consequently, it cannot assess causal relationships or track changes in anxiety and well-being as AI integration progresses. Future longitudinal studies are recommended to trace how students’ psychological adaptation develops across their academic and professional trajectories.

Second, data were collected through self-reported questionnaires, which may be subject to response and social-desirability biases. Triangulating these findings with qualitative interviews or behavioral observations would strengthen the validity and richness of future analyses. Third, although the sample of 820 students across four universities provides a strong foundation, it limits generalizability to other institutions, disciplines, and national contexts. Expanding future samples to include private universities, non-STEM majors, and international comparisons would provide a broader understanding of AI anxiety across different educational ecosystems.

In addition, the current study did not explicitly examine external influences such as socio-economic status, labor market volatility, or national AI policy frameworks, all of which may shape perceptions of career stability. Integrating these macro-level variables would provide a more holistic view of students’ experiences. Finally, the study’s focus on students excludes faculty and industry stakeholders, whose perspectives could illuminate systemic barriers between education and employment. Incorporating these viewpoints in future research would offer a more complete picture of how AI transformations affect the entire educational and professional pipeline.

## 6 Conclusion

This research highlights the urgent need for discipline-specific and gender-sensitive interventions to support students as they navigate AI’s accelerating influence on higher education and employment. By revealing the psychological mechanisms linking AI anxiety, digital well-being, and career concern, the study contributes to a nuanced understanding of how future professionals experience technological change. Universities and policymakers should therefore prioritize AI literacy programs that balance technical competence with emotional adaptability. Career resilience workshops and mental health initiatives can empower students—especially those in IT programs and female cohorts—to transform anxiety into preparedness and self-efficacy. Equally important, integrating ethical AI education into engineering and IT curricula will help students critically engage with the social, moral, and professional implications of automation. At the policy level, stronger university–industry partnerships and AI internship pathways can bridge the gap between education and the evolving labor market, fostering employability and confidence in AI-era professions. By proactively addressing these issues, Jordanian higher education institutions can cultivate an AI-literate, psychologically resilient, and socially responsible workforce—one capable of shaping, rather than fearing, the technological future.

## Data Availability

The datasets analyzed during the current study are not publicly available due to its confidentiality but are available from the corresponding author on reasonable request. Requests to access these datasets should be directed to m.alnasah@ju.edu.jo.
